# Calibration of Multi-Parameter Models of Avascular Tumor Growth Using Time Resolved Microscopy Data

**DOI:** 10.1038/s41598-018-32347-9

**Published:** 2018-09-28

**Authors:** E. A. B. F. Lima, N. Ghousifam, A. Ozkan, J. T. Oden, A. Shahmoradi, M. N. Rylander, B. Wohlmuth, T. E. Yankeelov

**Affiliations:** 1 Institute for Computational Engineering and Sciences, The University of Texas at Austin, Austin, 78712 USA; 2 Department of Mechanical Engineering, The University of Texas at Austin, Austin, 78712 USA; 3 Department of Biomedical Engineering, The University of Texas at Austin, Austin, 78712 USA; 4 Department of Neurology, Dell Medical School, The University of Texas at Austin, Austin, 78712 USA; 5 Department of Mathematics, Technical University of Munich, Garching, 85748 Germany; 6 Department of Diagnostic Medicine, The University of Texas at Austin, Austin, 78712 USA; 7 Department of Oncology, The University of Texas at Austin, Austin, 78712 USA; 8 Livestrong Cancer Institutes, Dell Medical School, The University of Texas at Austin, Austin, 78712 USA

## Abstract

Two of the central challenges of using mathematical models for predicting the spatiotemporal development of tumors is the lack of appropriate data to calibrate the parameters of the model, and quantitative characterization of the uncertainties in both the experimental data and the modeling process itself. We present a sequence of experiments, with increasing complexity, designed to systematically calibrate the rates of apoptosis, proliferation, and necrosis, as well as mobility, within a phase-field tumor growth model. The *in vitro* experiments characterize the proliferation and death of human liver carcinoma cells under different initial cell concentrations, nutrient availabilities, and treatment conditions. A Bayesian framework is employed to quantify the uncertainties in model parameters. The average difference between the calibration and the data, across all time points is between 11.54% and 14.04% for the apoptosis experiments, 7.33% and 23.30% for the proliferation experiments, and 8.12% and 31.55% for the necrosis experiments. The results indicate the proposed experiment-computational approach is generalizable and appropriate for step-by-step calibration of multi-parameter models, yielding accurate estimations of model parameters related to rates of proliferation, apoptosis, and necrosis.

## Introduction

There has been increasing interest in the development and application of mathematical models to describe the initiation, growth, and response of tumors to treatment^1,2^. To predict the spatiotemporal evolution of tumors, it is essential that these models be calibrated against relevant experimental data. The process of model calibration requires that a sequence of experiments be designed and executed in which one or more dependent variables are fully prescribed while others are allowed to vary until the difference between the model and the data is minimized according to a pre-defined error function^3–11^. Two areas of investigation that are central to this process are related to (1) the increasing interplay between experiment and theory, and (2) characterizing the experimental and computational uncertainties inherent in such efforts. Without a fuller understanding of these two issues the modeling can, at best, produce only qualitative descriptions of tumor growth and cannot generally be used as a basis for predicting, with precision, the outcomes of various therapies. In the present effort, we begin to address both of these limitations by designing a set of *in vitro* studies to systematically provide inputs for a general class of mathematical models.

The capability of computational models to accurately predict the complex and dynamic nature of tumor progression, intrinsic intra-tumoral heterogeneity, and spatial aspects of tumor cell migration requires acquisition of experimentally measured parameters to capture these phenomena with sufficient spatial and temporal resolution. Serial microscopy measurements provide a convenient system in which to address these issue as both high spatial and temporal resolution data can be acquired over a sufficiently large field-of-view to enable characterization of biological heterogeneity. There have been some previous efforts, with varying levels of complexity, in the mathematical formulation and experimental parameter estimation for such system. In particular, some efforts have focused on modeling a homogenous tumor with emphasis on prediction of the time dependent response to therapy^12^. For example, McKenna *et al*. developed and experimentally validated a coupled pharmacokinetic/pharmacodynamics model to predict the temporal response of a homogeneous cell population to a temporally-varying treatment time course. In this study, longitudinal fluorescence microscopy experiments were used to characterize the dynamics of doxorubicin uptake and population response to this drug in a variety of triple negative breast cancer cells lines^12,13^. Waclaw *et al*. modeled the influence of intra-tumor heterogeneity on the spatial growth and resistance to therapeutic agents of hepatocellular carcinoma using neoplastic and non-neoplastic cells. Histopathology and microscopy were utilized to determine heterogeneous cell populations from human biopsies and this information provided critical input data for model formulation^14^. Tyson *et al*. modeled single cell proliferation and drug response of lung cancer cells. For these studies, immunofluorescence labeling and time-lapse automated microscopy were used to calibrate their model to estimate single cell lifespan^15^. Thus, there is a developing (and promising) field at the interface of time resolved microscopy data and mathematical modeling that should be extended, and for which the inherent experimental and model uncertainties must be quantified.

Mathematical models which represent physical phenomena are subject to the inherent uncertainties in both the experimental data and the models themselves. Experimental data can be corrupted by both random and systemic error which leads to uncertainty when using these data to populate and calibrate model parameters, determine domain geometry, and setting initial conditions. The mathematical models contribute uncertainty through inadequacies in their underlying biological and physical assumptions, and implementation of the boundary conditions. If such uncertainties are not accounted for, the model will provide predictions that are biased to an extent determined by those uncertainties. In the case of tumor modeling, these errors may lead to predictions that mischaracterize the location and extent of disease (global errors), and intra-tumoral heterogeneity (local errors). We have developed a general framework for handling these uncertainties based on Bayesian statistical inference^16,17^. Briefly, observational data are collected and given a prior probability density on the model parameters from which we compute the posterior probability density (i.e., the calibration step). Then using the posterior probability density obtained at the calibration step as a prior, we update the parameters for a different scenario, or with data not used during the calibration step (i.e., the validation step). The updated parameters are then used to solve the forward problem to determine prediction accuracy using an appropriate metric. Using this Bayesian approach, the parameters, the data, and even the model are not assumed to be deterministic; rather, they are considered as random variables characterized by probability density functions. These characterizations lead to a stochastic model that is able to propagate uncertainty from model inputs to outputs.

In this study, we describe a general approach for the Bayesian calibration of phenomenological models of avascular tumor growth that accounts for uncertainties in observational data, in model parameters, and in the prediction of principal quantities of interest (e.g., the growth or decline of the tumor over time). We use this methodology to provide a general approach for the calibration of multi-parameter models to time-resolved (*in vitro*) microscopy data in which the proliferation and death of cell colonies are controlled and measured for prescribed levels of available nutrients, and in which specific experimental protocols are designed to control, isolate, and monitor the evolution of (in this case) liver cancer cells. The design of an integrated experimental-computational framework, and the determination of uncertainties in model parameters are the main contributions of this work.

Following this introduction, we provide a brief review to a large class of phenomenological tumor growth models based on a combination of biological models of cell proliferation, hypoxia, necrosis, and balance laws of continuum mixture theory. These classes include reaction-diffusion models, phase-field models, and models which account for mechanical deformation effects on cell mobility. To characterize uncertainties in the observable data, in model selection, as well as in the stochastic nature of the tumor growth and the model parameters, we call upon Bayesian statistical calibration procedures. Here Markov Chain Monte-Carlo sampling methods are used to calculate the posterior probability densities of key model parameters. We then provide the experimental details which allow for model calibration of cell proliferation, cell death mechanisms, and mobility, as well as the influence of various concentrations of nutrient, and therapeutic agents. Finally, we provide detailed accounts of the statistical calibration of the *in vitro* experiments, and present probabilistic characterization of the key parameters.

## Materials and Methods

### Classes of phenomenological models of tumor growth

The class of tumor growth models considered here is an extension of the avascular model developed in^16,17^ in which we incorporate different phenotypes. Previously, we developed a system of coupled, nonlinear partial differential equations describing a 10-field, multispecies tumor growth model which accounted for proliferative, hypoxic, necrotic, healthy, and endothelial cells, as well as vascular endothelial growth factor and nutrients^3^. Here, we consider an open bounded region ${\rm{\Omega }}\subset {{\mathbb{R}}}^{2}$, with smooth boundary, $\partial {\rm{\Omega }}$, which contains an evolving and interacting mass of tumor cells concentrations of which are represented by scalar-valued functions of position $x\in {\rm{\Omega }}$ and time $t\in \mathrm{[0,}\,T]$, representing the volume fraction of each tumor cell species. The volume fraction of tumor cells at *x* at time *t* is denoted by ${\phi }_{T}(x,\,t)$, while that of proliferative, hypoxic, and necrotic cells is denoted ${\phi }_{P}(x,\,t)$, ${\phi }_{H}(x,\,t)$ and ${\phi }_{N}(x,\,t)$, respectively. To align the model complexity with experimental data, we denote the viable and tumor cells volume fraction by

1 $$\begin{array}{rcl}{\phi }_{V}(x,\,t) & = & {\phi }_{P}(x,t)+{\phi }_{H}(x,t),\\ {\phi }_{T}(x,\,t) & = & {\phi }_{V}(x,t)+{\phi }_{N}(x,t\mathrm{).}\end{array}\}$$

In general, the mass-balance equations for the mixture take the form,

2 $$\frac{\partial {\rho }_{\alpha }{\phi }_{\alpha }}{\partial t}+\nabla \cdot ({\rho }_{\alpha }{\phi }_{\alpha }{v}_{\alpha })={\rho }_{\alpha }(\nabla \cdot {J}_{\alpha }+{S}_{\alpha }),$$

where *v*_*α*_ is the local convection velocity of species *α*, ${\rho }_{\alpha }\,{J}_{\alpha }$ is the mass flux, ${\rho }_{\alpha }{S}_{\alpha }$ is the mass supplied constituent *α* by other constituents, $\nabla ={\sum }_{i}\frac{\partial }{\partial {x}_{i}}$ is the spatial gradient operator. A common assumption, and one invoked here, is that the mass densities *ρ*_*α*_ of all constituents are essentially the same *ρ*_*α*_ = *ρ*_*c*_ = one constant^6,7,16–19^. Assuming that the evolution of tumor growth generally takes place over a relatively long time intervals, the convective velocity *v*_*α*_ can be set to zero, leaving the reduced form of mass balance,

3 $$\frac{\partial {\phi }_{\alpha }}{\partial t}=\nabla \cdot {J}_{\alpha }+{S}_{\alpha },\,\alpha =T,\,V,\,N\mathrm{.}$$

Various biological events (cell mitosis and proliferation, apoptosis, hypoxia, etc.) are modeled through fluxes and sources (i.e., *J*_*α*_ and *S*_*α*_, respectively) designed to follow rules inspired by well-known hallmarks of cancer cell biology. The mass flux *J*_*α*_ depends linearly on the gradient of the chemical potential *μ*, according to

4 $${{J}}_{\alpha }=-\,{{M}}_{\alpha }(\phi )\nabla \mu ,$$

where *M*_α_(*ϕ*) is the positive-semi-definite mobility matrix for species *α*, possibly dependent on species *ϕ*, *ϕ* = {*ϕ*_*T*_, *ϕ*_*V*_, *ϕ*_*N*_}.

The segregation and adhesion properties among the mixture constituents are modeled by a free energy functional (*E*) that sums the contribution of the *α*^th^ constituent, *E*_*α*_, to the total energy; that is, $E={\sum }_{\alpha =1}^{N}{E}_{\alpha }$. We assume that tumor cells prefer to adhere to each another^19^ so that they remain segregated, forming clusters of tumor cells, and conform to a double-well form of bulk energy, ${\rm{\Psi }}({\phi }_{T})$. The double-well function is zero at the pure phases, ${\phi }_{T}=0$ and ${\phi }_{T}=1$, driving the segregation into the pure phases. We also assume, for simplicity, that the adhesive properties of all tumor cells (viable and necrotic) are similar. As the adhesion between the cells increases, the length of the interface, *ε*_*T*_, between the pure phases decreases. These assumptions yield the following total energy of the system:

5 $$E={\int }_{\Omega }[{\rm{\Psi }}({\phi }_{T})+\frac{{\varepsilon }_{T}^{2}}{2}{|\nabla {\phi }_{T}|}^{2}]\,dx,$$

where *ε*_*T*_ is the interaction length (i.e., the boundary-layer thickness between phases), and ${\rm{\Psi }}(\phi )$ is defined by a quartic double-well potential. The double-well potential is assumed to be of the polynomial form,

6 $${\rm{\Psi }}({\phi }_{T})={\bar{E}}_{T}{\phi }_{T}^{2}{(1-{\phi }_{T})}^{2},$$

where ${\bar{E}}_{T} > 0$ is the energy scale associated with the tumor volume fraction. The free-energy can be decomposed into contractive and expansive terms^20^,

7 $${\rm{\Psi }}(\phi )={\bar{E}}_{T}{\phi }_{T}^{2}{\mathrm{(1}-{\phi }_{T})}^{2}={{\rm{\Psi }}}_{c}({\phi }_{T})-{{\rm{\Psi }}}_{e}({\phi }_{T}),$$

where

8 $$\begin{array}{cc}{{\rm{\Psi }}}_{c}({\phi }_{T})=\frac{3}{2}{\bar{E}}_{T}{\phi }_{T}^{2}, & {{\rm{\Psi }}}_{e}({\phi }_{T})=-\,{\bar{E}}_{T}({\phi }_{T}^{4}-2{\phi }_{T}^{3}-\frac{1}{2}{\phi }_{T}^{2})\mathrm{.}\end{array}$$

Such splitting, though not unique, always exists for each form of the energy functional and it provides a basis for developing unconditionally stable numerical schemes to advance the evolution of each cell species in time.

From arguments on the thermodynamics of the mixture (e.g.^9,10^), it can be inferred that the chemical potential is the partial Gateaux derivative of the total free energy *E*, with respect to the tumor volume fraction, *ϕ*_*T*_. Thus, for *E* given by Eq. (5),

9 $$\mu ={\rm{\Psi }}^{\prime} ({\phi }_{T})-{\varepsilon }_{T}^{2}{\rm{\Delta }}{\phi }_{T}\mathrm{.}$$

The mass source terms *S*_*α*_ in Eq. (3) are designed to capture various states of cell viability that depend on nutrient supply and cell concentrations. With the notion that the local dynamics of the tumor growth depend on the nutrient availability, *σ*_*VN*_ is introduced as the nutrient threshold which determines the transfer from viable to necrotic cell states. The viable tumor cells can grow until reaching the carrying capacity, *K*, when consuming nutrient with a constant rate of cellular mitosis, *λ*_*prol*_. They also decay owing to natural death of cells at the apoptosis rate, *λ*_*apop*_. Moreover, when the level of nutrient drops below *σ*_*VN*_, the viable cells become part of the necrotic core at the rate, *λ*_*VN*_. We assume that the necrotic core of the tumor never decreases. With these assumptions, the source terms in our model take the following forms:

$${S}_{V}={\lambda }_{prol}{\phi }_{\sigma }{\phi }_{V}(1-\frac{{\phi }_{V}+{\phi }_{N}}{K})-{\lambda }_{apop}{\phi }_{V}-{\lambda }_{VN} {\mathcal H} ({\sigma }_{VN}-{\phi }_{\sigma }){\phi }_{V},$$

$${S}_{N}={\lambda }_{VN} {\mathcal H} ({\sigma }_{VN}-{\phi }_{\sigma }){\phi }_{V},$$

where ${\mathcal H}$ is the Heaviside step function (${\mathcal H} (x)=0$, $x < 0$, ${\mathcal H} (x)=1$, $x\ge 0$).

In light of the above arguments, assumptions, and approximations, the governing equations of the model areIn Eq. (10), the mobility of the necrotic cells is disregarded as these cells are dead due to the lack of sufficient nutrients. Consequently, the mobility of the tumor cells is defined as

11 $${\bar{M}}_{T}(\phi )=\{\begin{array}{ll}{M}_{T} & {\rm{if}}\,{\phi }_{T}=\mathrm{0,}\\ {M}_{T}(1-\frac{{\phi }_{N}}{{\phi }_{T}}) & {\rm{otherwise}}{\rm{.}}\end{array}$$

The necrosis nutrient threshold, ${\sigma }_{VN}$, is determined experimentally following the 10% fetal bovine serum “optimal” value, as provided by the vendor (American Type Culture Collection, ATCC). Thus, seven model parameters remain to be determined or calibrated (highlighted in Eq. (10)). To solve these equations, appropriate boundary and initial conditions are required and we take zero flux boundary conditions on ${\phi }_{T}$, ${\phi }_{N}$, and *μ* (see e.g.^6,16,17^):

12 $$\nabla {\phi }_{T}\cdot n=\mathrm{0,}\,\nabla {\phi }_{N}\cdot n=\mathrm{0,}\,\nabla \mu \cdot n=\mathrm{0,}\,{\rm{on}}\,\,\partial {\rm{\Omega }}\times \mathrm{(0,}T),$$

where *n* is normal to $\partial {\rm{\Omega }}$. The initial conditions $0\le {\phi }_{T0}(x)\le 1$ and $0\le {\phi }_{N0}(x)\le 1$, $\forall \,x\in {\rm{\Omega }}$, are measured directly by the experiments described below.

### Bayesian statistical calibration

Bayesian inversion provides a comprehensive and consistent framework for statistical calibration of model parameters which accounts for uncertainties in both observational data and in model selection, as well as delivering consistent probabilistic representations of model parameters^3,17,21^. We now summarize the key features of our Bayesian parameter estimation (please see the Supplementary Material for more details).

Let $A:{\rm{\Theta }}\times U\to V$ denote the operators defining the model of interest, *U* and *V* being appropriate function spaces, and let the abstract problem of finding $\phi \in U$ such that,

13 $$A(\theta ,\phi )=\mathrm{0,}$$

be the *forward problem*, $\theta \in {\rm{\Theta }}$ being a vector of model parameters taken from a parameter space ${\rm{\Theta }}$. For the problem described by Eq. (13), $\theta ={\{{M}_{T},{\lambda }_{prol},K,{\lambda }_{apop},{\bar{E}}_{T},{\varepsilon }_{T},{\lambda }_{VN}\}}^{T}$,

14 $$A=\{\begin{array}{ll}\frac{\partial {\phi }_{T}}{\partial t} & \\ \mu  & -B(U)\\ \frac{\partial {\phi }_{N}}{\partial t} & \end{array},$$

$U={\{{\phi }_{T},\mu ,{\phi }_{N}\}}^{T}$, with $B(\,\cdot \,)$ the right-hand-side, and with the understanding that nutrient volume fraction ${\phi }_{\sigma }$ will be prescribed as data. The goal is to choose the parameters *θ* so that the model (13) agrees with a physical reality of interest denoted as *g*. Of course, such realities cannot be realized exactly; they are only available to use via experimental data *y* which is always corrupted by experimental noise.

Since the dependent variables in our model are volume fractions of different tumor cell phenotypes, and since the key quantities of interest could be chosen to be the viable tumor volume at times ${t}_{i}\in \mathrm{[0,}\,{T}_{end}]$, $i=\mathrm{1,}\,\mathrm{2,}\,\ldots ,\,n$, the physical realities of interest are ${g}_{i}={\mathbb{E}}[{\int }_{{\rm{\Omega }}}{\phi }_{V}(x,\,{t}_{i})dx]$ for the scenarios where the cellular spatial distribution is not available, and ${g}_{i}={\mathbb{E}}[{\phi }_{V}(x,\,{t}_{i})]$ otherwise, with ${\phi }_{V}={\phi }_{T}-{\phi }_{N}$. Thus, an experimental approach is needed that allows the measurement of ${\phi }_{V}$ at specified time intervals. The observational data $y={\{{y}_{i}\}}_{i=1}^{n}$ must, in these scenarios, consist of measurements of cell concentrations (or volume fractions) at these times given specified nutrient concentrations. (We describe in the next section the experimental procedure for measuring ${\phi }_{V}$, given ${\phi }_{\sigma }$.) The cell counts are actually estimated from fluorescence images (details provided below). The conversion from florescence signal to cell numbers (and, hences, volume fraction) includes experimental error or noise. We shall employ as a model of experimental noise the additive relation

15 $${g}_{i}={y}_{i}+{\varepsilon }_{i},\,{\varepsilon }_{i} \sim {{\mathbb{P}}}_{\nu },$$

*g*_*i*_ being the physical reality at test point *i*, *y*_*i*_ the observational data, and ${{\mathbb{P}}}_{v}$ a probability model for *ε*_*i*_ with new hyperparameters (i.e., a parameter that is not part of the model under analysis) *v*. We employ a Gaussian noise model written as:

16 $${{\mathbb{P}}}_{\nu } \sim {\mathscr{N}}(m,{\sigma }^{2}I),$$

*m* being a mean, and ${\sigma }^{2}$ the variance.

Next we turn to the model prediction. For a sample $\theta \in {\rm{\Theta }}$ of the model parameters drawn from the parameter space ${\rm{\Theta }}$, we solve the forward problem (i.e., Eq. (10) or its observation Eq. (13)) for $U(\theta ;x,\,t)$ and compute the model predictions, denoted ${d}_{i}(\theta )$ of the data *g*_*i*_. The difference between reality and prediction for each choice of $\theta$ is the model inadequacy ${\eta }_{i}(\theta )$. As in (15), we introduce a probabilistic model for model inadequacy:

17 $$\{\begin{array}{l}{g}_{i}={d}_{i}(\theta )-{\eta }_{i}(\theta ),\,i=1,\,2,\,\cdots ,n,\\ {\eta }_{i}(\theta ) \sim {{\mathbb{P}}}_{\gamma } \sim {\mathscr{N}}(\hat{m},{\hat{\sigma }}^{2}I),\end{array}$$

where $\hat{m}$ and ${\hat{\sigma }}^{2}$ are additional hyperparameters, and a Gaussian probability model is again employed. Subtracting Eq. (17) from Eq. (15) gives

18 $$\{\begin{array}{l}{y}_{i}-{d}_{i}(\theta )={\varepsilon }_{i}+{\eta }_{i}(\theta ),\\ {{\mathbb{P}}}_{\nu +\gamma }\sim {\mathscr{N}}(m,{\sigma }^{2}I)+{\mathscr{N}}(\hat{m},{\hat{\sigma }}^{2}I).\end{array}$$

If ${{\mathbb{P}}}_{\nu +\gamma }$ is the probability density of the combined noise-inadequacy model (18), the likelihood probability density $\pi (\,\cdot \,,\,\cdot \,)$ is the conditional density

19 $$\{\begin{array}{rcl}\pi ({y}_{i}|\theta ) & = & {{\mathbb{P}}}_{\nu +\gamma }({\varepsilon }_{i}+{\eta }_{i}(\theta )),\\  & = & {{\mathbb{P}}}_{\nu +\gamma }({y}_{i}-{d}_{i}(\theta ),\end{array}$$

where $$i=\mathrm{1,}\,\mathrm{2,}\,\ldots ,\,n$$.

Since a Bayesian calibration process is to be used, prior probability densities $\pi (\theta )$ on all parameters must be specified. When no prior information is known about a parameter ${\theta }_{i}$, except bounds ${\theta }_{i}\in (a,b)$, we employ the uniform prior ${\theta }_{i} \sim {\mathscr{U}}(a,\,b)$. If mean and variance are known approximately, a Gaussian prior, or a truncated Gaussian, can be used. The Bayesian update in model parameters *θ* is furnished by the posterior probability density $\pi (\theta |{\bf{y}})$

20 $$\pi (\theta |{y}_{i})=\frac{\pi ({y}_{i}|\theta )\pi (\theta )}{\pi ({y}_{i})},$$

where the denominator, called the model evidence, is the normalizing factor

21 $$\pi ({y}_{i})={\int }_{{\rm{\Theta }}}\pi ({y}_{i}|\theta )\pi (\theta )d\theta \mathrm{.}$$

Based on the assumptions of a Gaussian noise-inadequacy model and independent and identically distributed samples of experimental data, we then construct the likelihood function (19) as:

22 $$\pi (y|\theta )=\prod _{j=1}^{{{\mathscr{N}}}_{t}}\prod _{i=1}^{{{\mathscr{N}}}_{r}}\frac{1}{\sqrt{2\pi {\sigma }^{2}}}{e}^{-\frac{{({y}^{ij}-{d}_{c}^{j}(\theta ))}^{2}}{2{\sigma }^{2}}},$$

where ${{\mathscr{N}}}_{r}$ is the number of days measured, ${{\mathscr{N}}}_{r}$ is the number of replicates per day, $\sigma$ is an hyperparameter (the standard deviation for the Gaussian) related to the size of the noise, ${d}_{c}$ is the volume fraction of viable cells predicted by the model, and *y* is the experimentally measured data against which the model is calibrated. In Eq. (22), ${y}^{ij}$ is ${\phi }_{V}^{ij}$ at well i $1\le i\le 4$, and $j$ is the day of the measurement.

For the scenarios where the spatial distribution must be taken into account, such as the mobility calibration, the Pearson correlation coefficient (PCC) is computed to measure the linear correlation between the measured data and the calibrated model. The viable tumor volume fraction from each element of the discretized computational domain is compared with its correspondent position in the measured data. The PCC has a value between 1 and −1, indicating total positive linear correlation or total negative linear correlation, respectively. A zero PCC indicates that there is no linear correlation between the two. As we change the mobility of the tumor cells, a different distribution of the tumor cell volume fraction will be found, which can increase or decrease the correlation between the model solution and the experimental data. We modify the likelihood function in Eq. (22) to take into account the PCC information. To maximize the linear correlation (i.e., PCC equals one), we construct the following optimization function $\gamma (y|\theta )$ defined as:

23 $$\gamma (y|\theta )=\prod _{j=1}^{{{\mathscr{N}}}_{t}}\prod _{i=1}^{{{\mathscr{N}}}_{r}}\frac{1}{\sqrt{2\pi {\sigma }^{2}}}{e}^{-\frac{{\mathrm{(1}-PCC({y}^{ij},{d}_{c}^{j}(\theta )))}^{2}}{2{\sigma }^{2}}}\mathrm{.}$$

In the next section we describe the experiments designed to provide the probability densities of the model parameters. The forward problem given by Eq. (10) is solved for *M* samples of the probability densities of each parameter using a Monte Carlo algorithm^22^.

### Microscopy

Serial microscopy was performed using two different microscopes in this study. A BioTek plate reader (BioTek, Winooski, VT) was used to measure fluorescence of CellTiter-Blue assay with response to reaction with viable cells every 24 hours. In this assay, metabolic active cells produce fluorescent products in the media which is proportional to the number of viable cells. The produced dye is homogeneously distributed in each sample. A single measurement is collected from each well as a representative measurement of the whole sample. For each measurement, there were four biological replicates. A Leica SP8 confocal microscope (Leica, Wetzlar, Germany) was used to capture the number and spatial distribution of cells within each well during each experiment described below. Each well was imaged at 30 minute intervals *via* fluorescence microscopy with a 10X dry objective in 1.16 mm × 1.16 mm image montages with 2.275 *μ*m pixel size.

#### Cell culture preparation

Liver cancer, C3Asub28, cells were generously donated by Dr. Wei Li (The University of Texas at Austin) and cultured with DMEM/F12 (1:1) + L-Glutamine, +15 mM HEPES (Invitrogen, CA) supplemented with 10% fetal bovine serum (FBS, Sigma Aldrich, MO) and 1% Penicillin/Streptomycin (Invitrogen, CA). Cells inside T-flasks were incubated (Thermo, MA) at 37 °C, 95% atmospheric air (5% CO_2_). Cell confluence was monitored every day and cells were used in experiments only when they were 70% confluent. The cells were then detached from the flasks and seeded to a 96-well plate (Corning Life Sciences, Cambridge, MA) with desired seeding density for further analysis. Samples were incubated at 37 °C, 95% atmospheric air (5% CO_2_) and media was replaced daily. All experiments and time points were biologically replicated for 4 times to estimate statittical significance and repeatability.

#### Determination of growth rate and cell viability

Cell viability was assessed using CellTiter-Blue® assay (Promega, WI) following the manufacturer’s protocol. Viability of cells with 5 different initial seeding densities (5.0 × 10^3^, 1.0 × 10^4^, 2.5 × 10^4^, 5.0 × 10^4^, and 1.0 × 10^5^ cells/ml) were tested every 24 hours for 21 days. Before applying the assay and measuring viable cell counts, cells were rinsed twice with phosphate-buffered saline (PBS, Sigma Aldrich, MO) to remove waste generated by the cells, which can affect the results of the assay due to increased acidity of the media.

#### Determination of apoptosis rate

Apoptosis rate is commonly measured by labeling apoptotic cells with appropriate marker and counting by using flow cytometry. An alternative method to measure apoptosis rate is shown as treating cells with decent concentration of Mitomycin C (MMC, Sigma Aldrich, MO). MMC is known as an inhibitor of cell proliferation^23,24^ and 10 *μ*g/ml MMC treatment has shown to inhibit proliferation accordingly previous studies^25,26^. Therefore, to measure apoptosis rate within the same experimental setup, MMC treatment method was used. Cells were treated with 10 *μ*g/ml MMC for 120 minutes before detaching from the culture flask. After this treatment, MMC treated cancer cells were detached from culture flask and seeded with five different initial densities (5.0 × 10^3^, 1.0 × 10^4^, 2.5 × 10^4^, 5.0 × 10^4^, and 1.0 × 10^5^ cells/ml) to 96 well plates. The viability protocol described in the previous section was conducted every 24 hours for 7 days. As the cell proliferation is inhibited, and the cells are in a nutrient-rich environment (i.e., 10% concentration of FBS), the only phenomena driving the change in the measured viability of cells is the apoptosis rate. These viability curves are used to calibrate the apoptosis rate from the model in Eq. (10).

#### Determination of growth rate with varying levels of nutrient concentration and necrosis rate

To investigate the effects of varying levels of nutrient concentration on the viability of cells, the concentration of FBS in the cell media was decreased from the usual concentration of 10% to 7.5%, 5%, 2.5% and 0% in a set of independent experiments. Four different initial cell concentrations were also utilized: 1.0 × 10^4^, 2.5 × 10^4^, 5.0 × 10^4^, and 1.0 × 10^4^ cells/ml to determine the impact on growth rate. Viability measurements were performed every 24 hours for 7 days. From the same set of samples, the cell death rate due to necrosis (primarily due to a lack of nutrient availability) can be also quantified. For the calibration of this scenario, the apoptosis rate is considered to be known from the previous experiments. Therefore, the increase in cell death is considered to be a consequence of cell starvation due to the change of FBS concentration. This parameter in Eq. (10), the necrosis rate (${\lambda }_{VN}$), is calibrated using the viability curves measured with different FBS concentrations.

#### Determination of cell mobility with varying levels of nutrient concentration

Cell mobility in natural growth conditions were conducted by tracking each individual cell located in a 96 well plate. The C3Asub28 cells were transfected with green fluorescent protein (GFP) to label and allow for cell tracking. Cell locations were measured every 30 minutes for 12 hours for a initial seeding densities of 5.0 × 10^4^ cells/ml, and 5 different nutrient concentrations (10%, 7.5%, 5%, 2.5% and 0%). Cell seeding density of 5.0 × 10^4^ was selected for mobility experiments, since 5.0 × 10^4^ is the optimal seeding density for this cell type. The cells are segmented in MATLAB through a color-based segmentation using k-means clustering.

#### Conversion of fluorescence intensity measurements to cell number

The conversion of fluorescence intensity to cell number is essential to obtain estimates of the physical parameters included in the mathematical model. A proxy measure of cell volume fraction can be obtained by performing a linear correlation between the initial cell count on day 0 with its fluorescence intensity. This allows an estimation of the number of cells, ${N}_{c}(t)$, which can then be divided by the area of the well to compute the viable tumor cells volume fraction ${\phi }_{V}(t)$. The cell diameter is assumed constant, as no intervention was done that would alter the cytoplasm of cell membrane to cause the cells to change size.

The resulting cell number versus time data is calibrated to each model described above to estimate model parameters. To access the quality of the calibration, a metric using the cumulative probability distribution functions in ${L}^{1}({\mathbb{R}})$ is proposed: Let ${F}_{t}({\phi }_{V})$ and ${S}_{t}({\phi }_{V})$ be the cumulative distribution functions for the model and the data at day t, respectively. The ${L}^{1}({\mathbb{R}})$ metric for the data is then

24 $${{\rm{d}}}_{t}({F}_{t},{S}_{t})=\frac{{\int }_{0}^{\infty }|{F}_{t}({\phi }_{V})-{S}_{t}({\phi }_{V})|\,{\rm{d}}{\phi }_{V}}{{\bar{y}}_{t}},$$

where ${\bar{y}}_{t}$ is the mean tumor volume fraction from the data at time *t*. The simulations are computed on the Lonestar 5 Cluster, at the Texas Advanced Computing Center (TACC) of The University of Texas at Austin (URL: http://www.tacc.utexas.edu). The general purpose finite-element library libMesh^27^, is used to compute the numerical solutions to the partial differential equations. The ordinary differential equations are solved using a fourth-order Runge-Kutta method. For model calibration, we employ a multilevel Monte Carlo algorithm^22^ that is available in the library QUESO (Quantification of Uncertainty for Estimation, Simulation, and Optimization)^28^. The code itself, as a well as a description of how to use it, is provided at https://github.com/eabflima/invitro-liver.

## Results

### Calibration of apoptosis rates

**Figure 1:**
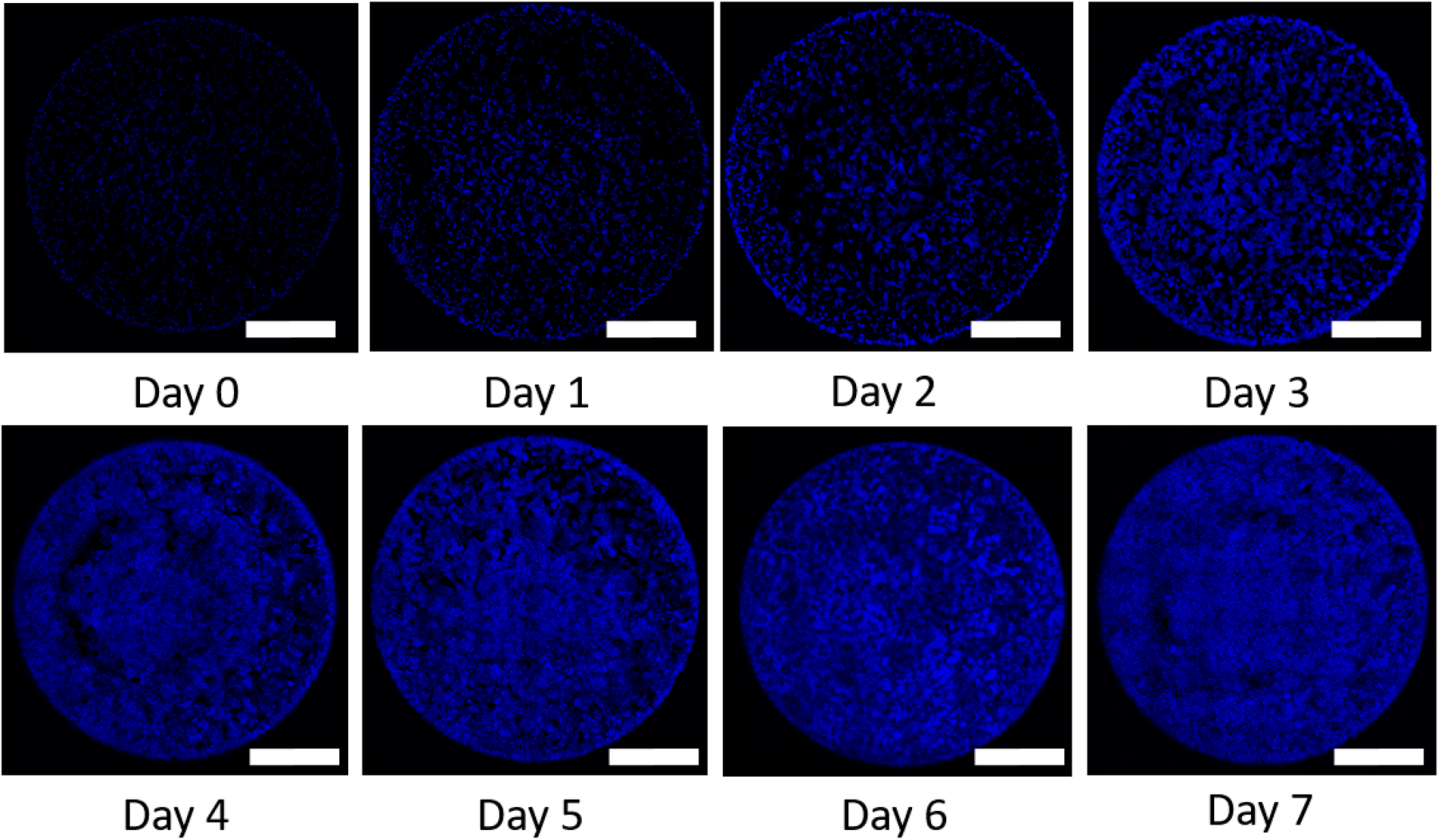
The panels show the temporal changes in cell confluence (stained with DAPI, blue) eight time points acquired over the period of 7 days indicating changes in cell confluence and spatial distribution. These are the data types that are used to calibrate the models. Scale is 1 mm.

**Figure 2:**
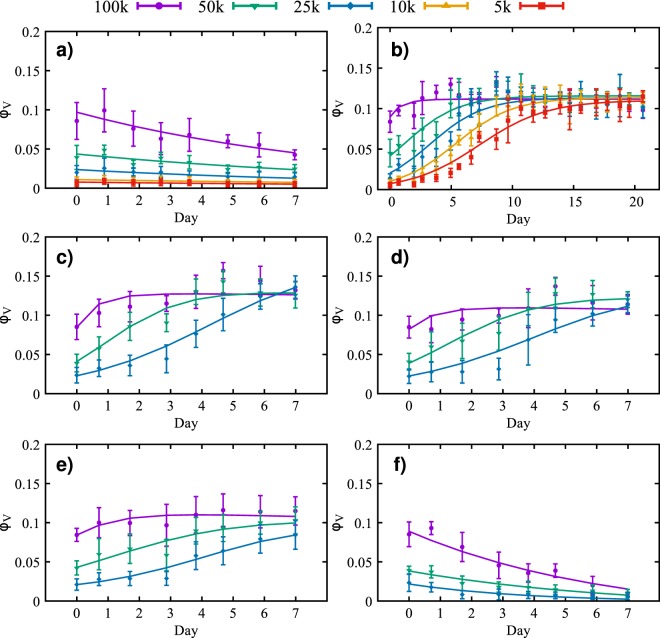
Time courses for the volume fraction (solid points) and credible interval (the vertical error bars) of viable tumor cell number for different initial cell concentrations: 100,000 (purple), 50,000 (green), 25,000 (blue), 10,000 (orange), and 5,000 (red) cells/ml. The solid lines represent the calibrated model. The data in panel (a) shows the effects of apoptosis after the cells are treated with 10 *μ*g/ml of Mitomycin-C for two hours. The average calibration error, for each initial condition, is between 11.54% and 14.04%. In panel (b) untreated cells in a serum concentration of 10% display an initial growth phase in which the cells reach the carrying capacity. The average calibration error, for each initial condition, is between 7.33% and 23.30%. In panels (c–f) the growth or decline of the tumor cells is observed for different concentrations of nutrient: 7.5% (**c**), 5% (**d**), 2.5% (**e**) and 0% (**f**). The maximum volume fraction decreases as the initial concentration of nutrient is reduced. The average calibration error, for each condition, is between 8.12% and 31.55%, with the highest error for the 25,000 cells/ml in (**f**).

Figure 1 displays serial microscopy images of C3A liver cancer cells (blue) over a period of 7 days. During this time the confluence clearly increases indicating that proliferation is occurring. To calibrate the apoptosis rate (${\lambda }_{apop}$), we stop cell proliferation by treating with Mitomycin-C, and acquire similar microscopy data (Fig. 2a). As we are only measuring the volume fraction of the viable tumor cells, initially without assessing the spatial distribution of the cells, and as the nutrient concentration is assumed to be sufficient to avoid hypoxia and necrosis, ${\phi }_{T}$ can be assumed to be equal to ${\phi }_{V}$ (recall Eq. (1)). With these assumptions, the model in Eq. (10) reduces to simply exponential death:

25 $$\frac{d{\phi }_{V}}{dt}=-\,{\lambda }_{apop}{\phi }_{V}\mathrm{.}$$

Here, the apoptosis rate (${\lambda }_{apop}$), the initial tumor volume fraction (${\phi }_{V0}$), and the standard deviation ($\sigma$) hyperparameter from Eq. (22) are all calibrated. The calibration priors are assumed to be uniform distributions with ${\lambda }_{apop} \sim {\mathscr{U}}\mathrm{(0,}\,\mathrm{10)}$, ${\phi }_{V0} \sim {\mathscr{U}}{\mathrm{(10}}^{-5},\,\mathrm{1)}$, and $\sigma  \sim {\mathscr{U}}\,{\mathrm{(10}}^{-5},\,\mathrm{20)}$. The model is calibrated for the five initial cell concentration: $5.0\times {10}^{3}$, $1.0\times {10}^{4}$, $2.5\times {10}^{4}$, $5.0\times {10}^{4}$, and $1.0\times {10}^{5}$ cells/ml. The mean initial volume fractions, ${\phi }_{V0}$, computed from the experiments are: $4.22\times {10}^{-3}$, $8.44\times {10}^{-3}$, $2.11\times {10}^{-2}$, $4.22\times {10}^{-2}$, and $8.44\times {10}^{-2}$, respectively.

**Figure 3:**
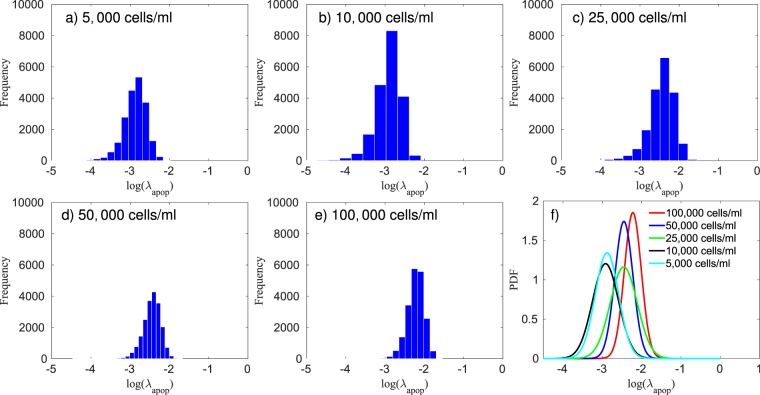
Histogram of the log of the apoptosis rate and the fitted probability density function (PDF) for five different initial conditions treated with Mitomycin-C at a concentration of 10 *μ*g/ml for two hours. The apoptosis rate is proportional to the initial concentration of cells, with the 5,000 and 10,000 cells/ml (panels a and b, respectively) having the lowest ($0.06\pm 0.03$), the 25,000 and 50,000 cells/ml (panels c and d, respectively) the intermediate ($0.09\pm 0.05$ and $0.09\pm 0.04$, respectively), and the 100,000 cells/ml (**e**) the highest apoptosis rate ($0.11\pm 0.05$).

**Figure 4:**
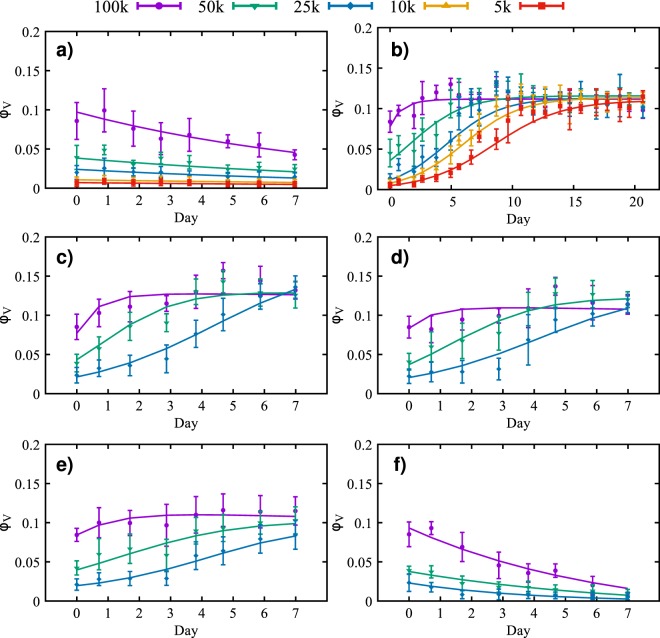
Time courses for the volume fraction (solid points) and credible interval (the vertical error bars) of viable tumor cell number for different initial cell concentrations: 100,000 (purple), 50,000 (green), 25,000 (blue), 10,000 (orange), and 5,000 (red) cells/ml. The solid lines represent the cross validation of the calibrated model. The data in panel (a) shows the effects of apoptosis after the cells are treated with 10 *μ*g/ml of Mitomycin-C for two hours. The average error, for each initial condition, is between 10.27% and 15.14%. In panel (b), untreated cells in a serum concentration of 10% display an initial growth phase in which the cells reach the carrying capacity. The average error for this case is between 7.25% and 15.24%. In panels (c–f) the growth or decline of the tumor cells is observed for nutrient concentrations of 7.5% (**c**), 5% (**d**), 2.5% (e) and 0% (**f**). The maximum confluence (i.e., the carrying capacity) decreases as the initial concentration of nutrient is reduced. The average error, for each condition, is between 8.35% and 35.02%, with the highest error for the 25,000 cells/ml in panel (f).

In Fig. 2a we compare the calibration of the model with the *in vitro* experiments. The 95% credible interval presented in Fig. 2a is determined by the lower and upper 2.5 percent points^29,30^. The solution of the forward model is obtained by sampling 200,000 values from the calibrated posterior of ${\lambda }_{apop}$, and averaging the output of the model, ${\phi }_{V}$. Using the metric given in Eq. (24), the average error per initial condition (5,000 to 100,000 cells/ml) is 11.98%, 11.54%, 14.04%, 13.30%, and 12.59%, respectively. In Fig. 3, the histogram of the apoptosis rate, ${\lambda }_{apop}$, for different initial conditions are presented. The results from the calibration indicates that the ${\lambda }_{apop}$ is proportional to the initial concentration of cells, with the 5,000 and 10,000 cells/ml having the lowest ($\approx {\mathscr{N}}\mathrm{(0.06,}\,\mathrm{0.02)}$), the 25,000 and 50,000 cells/ml the intermediate ($\approx {\mathscr{N}}\mathrm{(0.09,}\,\mathrm{0.03)}$, and ${\mathscr{N}}\mathrm{(0.09,}\,\mathrm{0.02)}$, respectively), and the 100,000 cells/ml the highest apoptosis rate ($\approx {\mathscr{N}}\mathrm{(0.11,}\,\mathrm{0.02)}$). In Table 1, we summarize the fitted distributions for the parameters. It is possible to observe that this concentration of Mitomycin-C is sufficient to stop cell proliferation. This conclusion can be made by observing the 95% equal-tail credibility interval (i.e., the range of values within which parameter values falls 95% of the time). In particular, the calibrated ${\lambda }_{apop}$ values are $0.06\pm 0.03$, $0.06\pm 0.03$, $0.09\pm 0.05$, $0.09\pm 0.04$, and $0.11\pm 0.05$ which correspond to the initial conditions of 5,000, 10,000, 25,000, 50,000, and 100,000 cells/ml, respectively. Note that a negative apoptosis rate would indicate that the cells were proliferating. In Table 2 we present the average error of the model prediction for the cross-validation experiments. The steps followed for the cross-validation experiments are: 1) calibrate the parameters using three out of the four replicates, 2) simulate the forward model 200,000 times using sampled values from the calibrated parameters, and the initial measurement from the replicate not used in step 1) as the initial condition, and 3) compute the error between the model-predicted and experimentally measured time course not used in step 1) using the metric defined in Eq. (24). The results of this procedure are displayed in Fig. 4.

**Table 1:** Fitted distributions for the calibrated parameters under different initial and FBS conditions.

Parameter	Initial condition (1,000 cells/ml)				
	5	10	25	50	100
*λ*_*apop*_ (day^−1^)	${\mathscr{N}}(0.06,0.02)$	${\mathscr{N}}(0.06,0.02)$	${\mathscr{N}}(0.09,0.03)$	${\mathscr{N}}(0.09,0.02)$	${\mathscr{N}}(0.11,0.02)$
*λ*_*prol*_ (day^−1^)	${\mathscr{N}}(0.43,0.02)$	${\mathscr{N}}(0.48,0.02)$	${\mathscr{N}}(0.55,0.05)$	${\mathscr{N}}(0.55,\mathrm{0.0.5})$	$\mathrm{ln}\,{\mathscr{N}}(0.43,0.64)$
*K* (dimensionless)	${\mathscr{N}}(0.13,0.01)$	${\mathscr{N}}(0.13,0.01)$	${\mathscr{N}}(0.14,0.01)$	${\mathscr{N}}(0.14,0.01)$	${\mathscr{N}}(0.12,0.01)$
*λ*_*VN*_ (day^−1^) (75% FBS)	n/a	n/a	$\mathrm{ln}\,{\mathscr{N}}(\,-\,5.20,1.22)$	$\mathrm{ln}\,{\mathscr{N}}(\,-\,5.94,1.21)$	$\mathrm{ln}\,{\mathscr{N}}(\,-\,6.31,1.22)$
*λ*_*VN*_ (day^−1^) (50% FBS)	n/a	n/a	$\mathrm{ln}\,{\mathscr{N}}(\,-\,4.82,1.30)$	$\mathrm{ln}\,{\mathscr{N}}(\,-\,5.76,1.27)$	$\mathrm{ln}\,{\mathscr{N}}(\,-\,5.71,1.26)$
*λ*_*VN*_ (day^−1^) (25% FBS)	n/a	n/a	$\mathrm{ln}\,{\mathscr{N}}(\,-\,5.02,1.48)$	$\mathrm{ln}\,{\mathscr{N}}(\,-\,5.18,1.32)$	$\mathrm{ln}\,{\mathscr{N}}(\,-\,5.74,1.18)$
*λ*_*VN*_ (day^−1^) (0% FBS)	n/a	n/a	$\mathrm{ln}\,{\mathscr{N}}(\,-\,2.11,0.35)$	$\mathrm{ln}\,{\mathscr{N}}(\,-\,2.23,0.21)$	$\mathrm{ln}\,{\mathscr{N}}(\,-\,2.43,0.30)$
*ε*_*T*_ (*μ*m) (10.0% FBS)	n/a	n/a	n/a	$\mathrm{ln}\,{\mathscr{N}}(3.18,1.04)$	n/a
*ε*_*T*_ (*μ*m) (7.5% FBS)	n/a	n/a	n/a	$\mathrm{ln}\,{\mathscr{N}}(3.34,1.21)$	n/a
*ε*_*T*_ (*μ*m) (5.0% FBS)	n/a	n/a	n/a	$\mathrm{ln}\,{\mathscr{N}}(3.58,1.01)$	n/a
*ε*_*T*_ (*μ*m) (2.5% FBS)	n/a	n/a	n/a	$\mathrm{ln}\,{\mathscr{N}}(3.07,0.97)$	n/a
*ε*_*T*_ (*μ*m) (0.0% FBS)	n/a	n/a	n/a	$\mathrm{ln}\,{\mathscr{N}}(3.41,1.24)$	n/a
*M*_*T*_ (*μ*m/day) (10.0% FBS)	n/a	n/a	n/a	$\mathrm{ln}\,{\mathscr{N}}(5.22,1.52)$	n/a
*M*_*T*_ (*μ*m/day) (7.5% FBS)	n/a	n/a	n/a	$\mathrm{ln}\,{\mathscr{N}}(5.14,1.50)$	n/a
*M*_*T*_ (*μ*m/day) (5.0% FBS)	n/a	n/a	n/a	$\mathrm{ln}\,{\mathscr{N}}(5.28,1.44)$	n/a
*M*_*T*_ (*μ*m/day) (2.5% FBS)	n/a	n/a	n/a	$\mathrm{ln}\,{\mathscr{N}}(4.95,1.61)$	n/a
*M*_*T*_ (*μ*m/day) (0.0% FBS)	n/a	n/a	n/a	$\mathrm{ln}\,{\mathscr{N}}(5.57,1.26)$	n/a
${\bar{E}}_{T}$ (dimensionless) (10.0% FBS)	n/a	n/a	n/a	${\mathscr{U}}(0.15,1.15)$	n/a
${\bar{E}}_{T}$ (dimensionless) (7.5% FBS)	n/a	n/a	n/a	${\mathscr{U}}(0.15,1.15)$	n/a
${\bar{E}}_{T}$ (dimensionless) (5.0% FBS)	n/a	n/a	n/a	${\mathscr{U}}(0.15,1.15)$	n/a
${\bar{E}}_{T}$ (dimensionless) (2.5% FBS)	n/a	n/a	n/a	${\mathscr{U}}(0.15,1.15)$	n/a
${\bar{E}}_{T}$ (dimensionless) (0.0% FBS)	n/a	n/a	n/a	${\mathscr{U}}(0.15,1.15)$	n/a

**Table 2:** Average error of the model prediction for the cross-validation experiments.

Calibration scenario	Initial condition (1,000 cells/ml)				
	5	10	25	50	100
Apoptosis	13.29%	10.27%	13.44%	15.14%	12.62%
Proliferation	15.03%	12.34%	15.24%	11.46%	7.25%
Necrosis (7.5% FBS)	n/a	n/a	10.96%	8.35%	10.17%
Necrosis (5.0% FBS)	n/a	n/a	21.82%	10.69%	12.34%
Necrosis (2.5% FBS)	n/a	n/a	15.31%	12.02%	9.36%
Necrosis (0.0% FBS)	n/a	n/a	35.02%	16.55%	23.07%

### Calibration of proliferation rates

To calibrate the proliferation rate (${\lambda }_{prol}$), we use the dataset of cell volume fraction without Mitomycin-C (Fig. 2b). Similar to the apoptosis calibration, the model in Eq. (10) can be simplified to:

26 $$\frac{d{\phi }_{V}}{dt}={\lambda }_{prol}{\phi }_{\sigma }{\phi }_{V}(1-\frac{{\phi }_{V}}{K})-{\lambda }_{apop}{\phi }_{V},$$

where the proliferation rate (${\lambda }_{prol}$), the carrying capacity (*K*), and the standard deviation ($\sigma$) hyperparameter from Eq. (22) are all calibrated. With this experiment, we update the distributions of the apoptosis rate (${\lambda }_{apop}$) and the initial tumor volume fraction (${\phi }_{V0}$) during the calibration. The nutrient concentration is assumed to be sufficient to maximize the growth rate (${\phi }_{\sigma }=1$). The calibration priors are assumed to be uniform distributions with ${\lambda }_{prol} \sim {\mathscr{U}}\mathrm{(0,}\,\mathrm{10)}$, $K \sim {\mathscr{U}}\mathrm{(0,}\,\mathrm{1)}$, and $\sigma  \sim {\mathscr{U}}{\mathrm{(10}}^{-5},\,\mathrm{20)}$, and the posteriors from the calibration using the data with Mitomycin-C are used as priors for ${\phi }_{V0}$ and ${\lambda }_{apop}$ (i.e., the posteriors from Fig. 3 are used as priors in this scenario).

**Figure 5:**
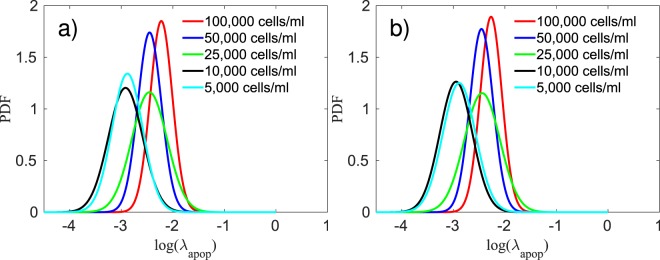
Panel (a) presents the posterior probability density function (PDF) of the log of the apoptosis rate obtained through the calibration of the data with Mitomycin-C. This PDF is used as a prior during the calibration of the proliferation rate for the data without Mitomycin-C. Panel (b) presents the posterior PDF of the log of the apoptosis rate computed during the calibration of the data without Mitomycin-C. The difference between the prior and posterior on the calibration of the apoptosis rate, from the lowest initial condition (5,000 cells/ml) to the highest (100,000 cells/ml), is 0.42%, 3.09%, 1.40%, 0.46%, and 4.38%, respectively. The small differences between the prior and posterior indicate that the apoptosis rate calibrated with Mitomycin-C is a good prior to calibrate the model without Mitomycin-C.

**Figure 6:**
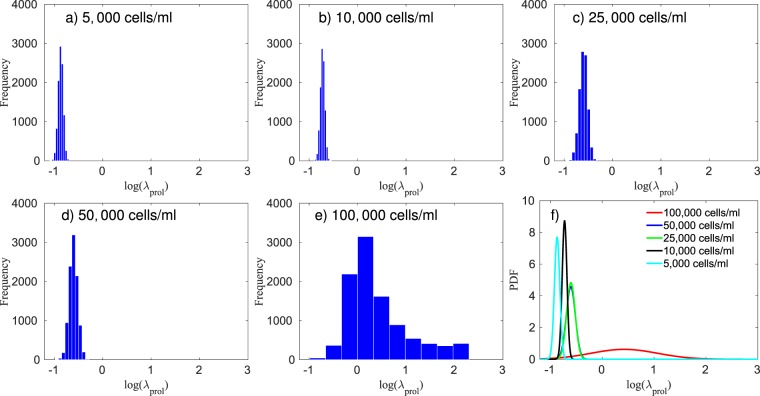
Panels (a–e) present the histogram of the log of the proliferation rate, ${\lambda }_{prol}$, for five different initial conditions, while panel (f) shows the fitted probability density function (PDF) for each of the cases. These data indicate the proliferation rate is proportional to the initial concentration of cells. The 95% equal-tail credibility interval for the proliferation rate, from the lowest initial condition (5,000 cells/ml) to the highest (100,000 cells/ml), is $0.42\pm 0.04$, $0.49\pm 0.04$, $0.55\pm 0.09$, $0.56\pm 0.09$, and $4.43\pm 3.75$, respectively. As the initial condition of the 100,000 cells/ml is close to the carrying capacity, the proliferation rate doesn’t affect the tumor growth after day 3, leading to a significantly wider range on the distribution of ${\lambda }_{prol}$.

In Fig. 2b, the calibrated model and the *in vitro* data are compared. To assess the quality of the calibration, we compute the average error per initial condition *via* Eq. (24). The errors, from the lowest initial condition (5,000 cells/ml) to the highest (100,000 cells/ml), are 23.30%, 15.56%, 19.46%, 12.13%, and 7.33%, respectively. In Fig. 5, we compare the prior and the posterior of the apoptosis rate. A metric similar to that in Eq. (24) is used with ${F}_{t}({\phi }_{V})$ and ${S}_{t}({\phi }_{V})$ now distribution functions for the posterior and the prior, and $\bar{y}$ the mean value of ${\lambda }_{apop}$ from the prior. The difference between the prior and posterior, per initial condition (5,000 to 100,000 cells/ml), on the calibration of the apoptosis rate, is 0.42%, 3.09%, 1.40%, 0.46%, and 4.38%, respectively. These small differences indicate that the scenario with Mitomycin-C is able to provide a calibrated apoptosis rate that allows the calibration of the proliferation rate without needing it to be significantly altered. In Fig. 6, the calibrated proliferation rates are presented. The approximated distributions for ${\lambda }_{prol}$, from the lowest initial condition to the highest, are $\approx {\mathscr{N}}\mathrm{(0.43,}\,\mathrm{0.02)}$, $\approx {\mathscr{N}}\mathrm{(0.48,}\,\mathrm{0.02)}$, $\approx {\mathscr{N}}\mathrm{(0.55,}\,\mathrm{0.05)}$, $\approx {\mathscr{N}}\mathrm{(0.55,}\,\mathrm{0.0.5)}$, and $\approx ln{\mathscr{N}}\mathrm{(0.43,}\,\mathrm{0.64)}$, respectively. The difference in the proliferation rate distribution for 100,000 cells/ml is due to the fact that, for this initial condition, the experiment starts with a volume fraction close to the carrying capacity of the well, as we can see in Fig. 2b. After the third day, the volume fraction of tumor cells is not affected by the value of ${\lambda }_{prol}$, which explains the different distribution patterns.

### Calibration of necrosis rates

The next experiment aims to calibrate the necrosis rate, ${\lambda }_{VN}$, using the data obtained from wells provided with different nutrient concentrations (Fig. 2c–f). In these experiments, the cell viability is measured for a series of nutrient concentrations that can lead to necrosis. With these assumptions, the model in Eq. (10) can be simplified to:

27 $$\begin{array}{rcl}\frac{d{\phi }_{T}}{dt} & = & {\lambda }_{prol}{\phi }_{\sigma }({\phi }_{T}-{\phi }_{N})(1-\frac{{\phi }_{T}}{K})-{\lambda }_{apop}({\phi }_{T}-{\phi }_{N}),\\ \frac{d{\phi }_{N}}{dt} & = & {\lambda }_{VN}({\phi }_{T}-{\phi }_{N}\mathrm{).}\end{array}\}$$

Note that in Eq. (27), we disregard the Heaviside function as we have observed that, for any concentration of nutrients below 10% of FBS, the condition ${\sigma }_{VN}-{\varphi }_{\sigma }\,\mathrm{ > }\,0$ holds. We calibrate the standard deviation ($\sigma$) hyperparameter from Eq. (22) and the necrosis rate (${\lambda }_{VN}$), and update the distributions of the apoptosis rate (${\lambda }_{apop}$), initial tumor volume fraction (${\phi }_{V0}$), proliferation rate (${\lambda }_{prol}$), and carrying capacity ($K$). The calibration priors are assumed to be uniform distributions with ${\lambda }_{VN} \sim {\mathscr{U}}\mathrm{(0,}\,\mathrm{10)}$, and $\sigma  \sim {\mathscr{U}}{\mathrm{(10}}^{-5},\,\mathrm{20)}$, and the posteriors from the previous scenario (where the proliferation rate is calibrated) are used as priors for ${\phi }_{V0}$, ${\lambda }_{apop}$, ${\lambda }_{prol}$, and *K*. The experiments are done with 0%, 2.5%, 5%, and 7.5% of FBS, which are mapped into nutrient volume fractions (${\phi }_{\sigma }$) as 0, 0.25, 0.5, and 0.75, respectively. The growth term, the first term on the right-hand side in the equation for ${\phi }_{T}$ in Eq. (27), assumes a linear relationship between the tumor and nutrient concentration^18,31–35^.

**Figure 7:**
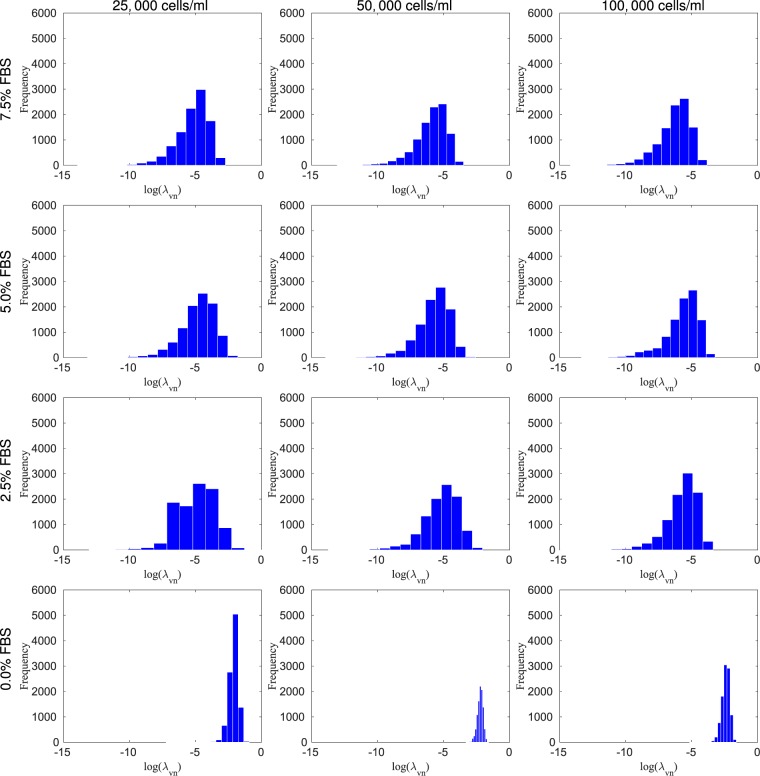
The histogram of the log of the necrosis rate, ${\lambda }_{VN}$, for four FBS concentrations of 7.5%, 5.0%, 2.5%, and 0.0%, from top to bottom, respectively, and three initial conditions, 25,000, 50,000, and 100,000 cells/ml, from right to left, respectively. The necrosis rate increases as the FBS concentration decreases. The panels presenting 2.5%, 5.0%, and 7.5% FBS indicate that there is little to no necrosis for these cases. It is only when the FBS level drops to zero, that evidence of necrosis appears.

In Fig. 2c–f the calibration of the model, at each nutrient condition, 7.5%, 5%, 2.5%, and 0% of FBS, respectively, is compared with the data from *in vitro* experiments. Using the metric given in Eq. (24), the errors, from the lowest initial condition to the highest, in this scenario 25,000, 50,000, and 100,000 cells/ml, and for the four nutrient concentration, are, respectively, c) 10.70%, 8.12%, and 10.06%; d) 20.87%, 9.93%, and 11.69%; e) 14.14%, 10.85%, and 8.46%; f) 31.55%, 14.90%, and 21.09%. In Fig. 7, the necrosis rate, ${\lambda }_{VN}$, for all combinations of initial nutrient and cell concentration are presented. We observe that ${\lambda }_{VN}$ increases as the nutrient available for the cells decreases.

### Calibration of tumor cell mobility

**Figure 8:**
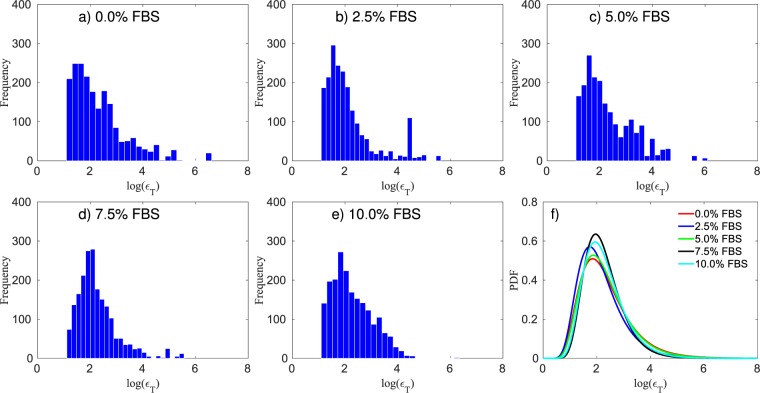
Panels (a–e) present the histogram of the log of the interaction length, ${\varepsilon }_{T}$, five different FBS concentrations (and the same initial cell density of 50,000 cells/ml), while panel (f) shows the fitted probability density function (PDF) for each of these cases. The 95% equal-tail credibility interval for the interaction length, from the lowest FBS (0.0%) to the highest (10.0%), is $94.54\pm 86.80$, $48.44\pm 45.25$, $47.91\pm 44.65$, $42.07\pm 38.53$, and $33.33\pm 30.07$, respectively.

**Figure 9:**
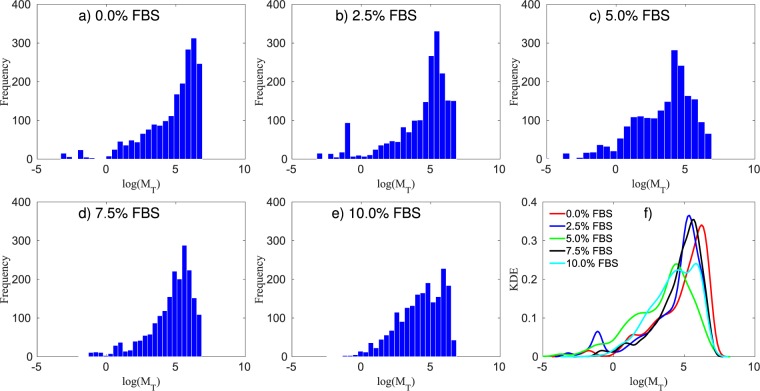
Panels (a–e) present the histogram of the log of the mobility rate, ${M}_{T}$, for five different FBS concentrations (and the same initial cell density of 50,000 cells/ml), while panel (f) shows the approximated probability density function (PDF) through the kernel density estimation. The 95% equal-tail credibility interval for the mobility rate, from the lowest FBS (0.0%) to the highest (10.0%), is $464.79\pm 463.83$, $370.00\pm 369.79$, $323.83\pm 323.60$, $370.34\pm 367.52$, and $293.36\pm 293.34$, respectively.

In this scenario, the tumor cells spatial distribution is measured, allowing us to calibrate the tumor mobility from the model in Eq. (10). As shown in Fig. 2a,b, there is minimal apoptosis and proliferation for the first 12 hours after the cells initial attach to the surface; thus, we disregard proliferation and apoptosis rates, and assume that necrotic cells are not present, leading to a simplified model:

28 $$\begin{array}{ccc}\frac{\partial {\phi }_{T}}{\partial t} & = & \nabla \cdot {\bar{M}}_{T}(\phi )\nabla \mu ,\\ \mu  & = & {\bar{E}}_{T}(2{\phi }_{T}-6{\phi }_{T}^{2}+4{\phi }_{T}^{3})-{\varepsilon }_{T}^{2}{\rm{\Delta }}{\phi }_{T}\end{array}\}$$

where the tumor mobility (${M}_{T}$), the energy scale (${E}_{T}$), the interaction length (${\varepsilon }_{T}$), and the standard deviation ($\sigma$) hyperparameter from Eq. (23) are all calibrated. The calibration priors are assumed to be uniform distributions with ${M}_{T}\sim {\mathscr{U}}\,\mathrm{(0,}\,\mathrm{1000)}$, ${E}_{T} \sim {\mathscr{U}}\,\mathrm{(0.15,}\,\mathrm{1.15)}$, ${\varepsilon }_{T} \sim {\mathscr{U}}\,\mathrm{(1,}\,\mathrm{1000)}$, and $\sigma  \sim {\mathscr{U}}{\mathrm{(10}}^{-5},\,\mathrm{0.2)}$. The experiments are done with $\mathrm{0 \% }$, $\mathrm{2.5 \% }$, $\mathrm{5 \% }$, $\mathrm{7.5 \% }$, and $\mathrm{10.0 \% }$ of FBS, and initial cell density of 50,000 cells/ml. In Fig. 8, we present the interaction length, ${\varepsilon }_{T}$, for the five FBS concentration. The approximated distributions for ${\varepsilon }_{T}$, for the five FBS concentrations (i.e., $\mathrm{0 \% }$, $\mathrm{2.5 \% }$, $\mathrm{5 \% }$, $\mathrm{7.5 \% }$, and $\mathrm{10.0 \% }$), are $\approx ln{\mathscr{N}}\mathrm{(3.41,}\,\mathrm{1.24)}$, $\approx ln{\mathscr{N}}\mathrm{(3.07,}\,\mathrm{0.97)}$, $\approx ln{\mathscr{N}}\mathrm{(3.58,}\,\mathrm{1.01)}$, $\approx ln{\mathscr{N}}\mathrm{(3.34,}\,\mathrm{1.21)}$, and $\approx ln{\mathscr{N}}\mathrm{(3.18,}\,\mathrm{1.04)}$, respectively. In Fig. 9, the mobility rate, ${M}_{T}$, for all initial FBS concentrations are presented. The energy scale ${\bar{E}}_{T}$ posterior remains the same as the prior; this is due to the configuration of the initial condition and the short time interval between the first and last measurement. The double-well potential is responsible for the separation of the mixture into pure constituents. As the initial condition used here consists of clusters of viable tumor cells, we do not observe the formation of new clusters during the duration of the experiments.

## Discussion

Mathematical models of malignant neoplasms are often developed either as a tool for improving the understanding of particular aspect of cancer biology; for example, understanding the underlying causes of tumor initiation and progression, or as tool for predictive modeling of therapeutic outcomes of cancer treatment. However, despite its relatively long history in cancer biology and oncology^36–38^, development and successful application of mathematical models of tumor growth is still a dauting challenge. This is largely due to the limited integration of theory with experiment, and the limited effort at characterizing the uncertainties in both the data and the models themselves. In this work, we used an extensive set of *in vitro* experiments to constrain and calibrate the parameters of a phenomenological, phase-field model of tumor growth. To calibrate the full phase-field model, we developed a hierarchical experimental design which allowed us to significantly reduce the complexity of the mathematical models to be calibrated. By applying state-of-the-art, multilevel Monte Carlo^28^ and hierarchical Bayesian techniques^21,39,40^, we were able to constrain the model parameters (i.e., proliferation, apoptosis, and migration) with experimentally available data.

The parameters and response kinetics obtained from our work could serve as the basis for pharmacokinetic/pharmacodynamics (PK/PD) modeling to measure the rate of apoptosis after treatment. By separating the contributions to cell death from apoptosis and necrosis, it is possible to differentiate apoptosis caused by a drug from that associated with natural cell death. Consequently, the apoptosis and necrosis rates in this study are key parameters for obtaining accurate PK/PD predictions. Also, it is known that cells experience intracellular signaling during proliferation^41^. Therefore, measuring and modeling cell mobility potentially enables us to predict interactions between cells during proliferation.

In comparing our parameter values to those previously reported, it is important to note that most previous efforts report apoptosis, proliferation, and necrosis as percentages rather than rates. This is accomplished experimentally (i.e., without calibrating data to a mathematical model) by computing the difference in the number of cells measured before, ${{\mathscr{N}}}_{1}$ and (for example) 24 hours after an intervention, ${{\mathscr{N}}}_{2}$, and dividing by ${{\mathscr{N}}}_{1}$. To compare our calibrated model with these experimentally-derived rates, we converted our model estimated rates to percentages by computing the difference in the simulated volume fraction before and after the same interval used experimentally. After this conversion, our apoptosis rates compare well to those previously reported for HEPG2 liver cancer cells. For example, Sun *et al*. showed that HEPG2 liver cancer cells have an apoptosis rate of 7.82 ± 1.29% per 24 hours based on their flow cytometry measurements^42^, whereas we found a value of 9.34 ± 1.21% per 24 hours at the same cell concentration (i.e., 100,000 cells/ml). Similarly, our proliferation rate showed variation between 19% and 42% before reaching the plateau-phase, while Wu *et al*. showed colorectal cancer cells exhibited a proliferation rate between 38% and 42%^43^. For necrosis, Kou *et al*. reported necrosis rates for HEPG2 liver cancer cells due to lack of nutrient as 33.87 ± 5.28%, which is similar to our finding of 29.01 ± 5.84%^44^. Interestingly, the cell mobility rate measured in our study was 27.55 ± 2.99 *μ*m/hour. Similarly, Zervantonakis *et al*. measured the cell mobility of HT100 cells (attracted to endothelial cells) as 29 ± 4.16 *μ*m/hour^45^, and Huang *et al*. reported MDA-MB-231 migration through softer tissues as 37 ± 2.72 *μ*m/hour^46^.

Calibration of model parameters requires complex experimental design that is able to simultaneously monitor the spatiotemporal evolution of (for example) tumor cell proliferation, viability, apoptosis rate, hypoxia, and mobility. This is, however, an extraordinarily difficult task given the currently available technologies. We therefore brought our calibration problem into the realm of experimental and computational feasibility by constraining and calibrating each parameter separately to data that acquired to specifically interrogate the parameter under investigation. One limitation inherent in this approach involves the estimation of the mobility parameter, *M*_*T*_. Importantly, when calibrating Eq. (28) to determine tumor cell mobility, we are required to use a shorter time series of microscopy data (approximately 12 hours) so as to separate observations of mobility from proliferation. However, our expression for the chemical potential, *μ* (the second equation in (28)), is also determined during the 12 hour time scale. This is a limitation because *μ* is dependent on *ϕ*_*T*_ which is clearly a function of time; but since *ϕ*_*T*_ is assumed to not change during these 12 hours, our characterization of *μ* may not be accurate and this may manifest itself as an source of error in the estimation of the mobility. More extensive experiments would be required to further separate these parameters. Importantly, the model has the capability to calibrate mobility for cells with more invasive phenotypes and, therefore, greater movement.

The calibration experiments and results described here are readily applicable to any family of models, and to scenarios for which the parameters in those models can be isolated experimentally for model calibration. In fact, there are a number of limitations in the present approach. In particular, the tumor microenvironment was not considered in these experiments. More specifically, the experimental systems used for model calibration consisted of a 2D cell monolayer of liver cancer cells which neglected the 3D extracellular matrix effects which are known to influence cell proliferation rates, cell-matrix interaction, and diffusion of nutrients. As such, key components of the extracellular matrix, including porosity, fiber diameter, pore size, or matrix stiffening^47–50^ that influence the transport of nutrients and, ultimately, cell proliferation and migration were not considered. This goal will be reached by implementing a tunable scaffold material such as collagen, fibrin or collagen-fibrin blend with suitable pH, temperature and representative compression modulus^48^. The lack of vasculature in our system discounted the influence of transport and distribution properties such as flow rate and shear stress, which are important for secretion of functional proteins as shown by vascularized *in vitro* study conducted by Ozkan *et al*.^48^. Introducing vasculature in the suggested scaffold material to the system can be done either by extractive needle or nanoimprinting methods as previously described by Michna *et al*. and Ozkan *et al*.^48,51^. The lack of endothelial cells within the matrix or vasculature eliminates the influence of angiogenesis and cancer-endothelial interaction, which is an important aspect of tumor growth^52^. Primary human umbilical vein endothelial cells (HUVEC) will be seeded around the created vasculature to form a more representative system and functionality of the vessel will be tested by measuring permeability and comparing with *in vivo* findings with different nanoparticle sizes. Additionally, vessel porosity will be determined by transfecting endothelial cells with appropriate markers and measuring vessel confluency with confocal microscopy to implement in the computational flow model. Furthermore, the lack of stromal cells such as fibroblast in the system limits the influence of remodeling of the extracellular matrix as the tumor grows and migrates^47^. Lastly, co-culture of Stella and Kupffer cells with hepatocellular carcinoma cells will improve representability of the complex liver carcinoma model and improve drug treatment response. Even though the current scenarios might be a tremendous simplification of the *in vivo* scenario, the approach used here can readily provide a more informed prior than assuming a non-informative prior (e.g., a uniform distribution) for subsequent *in vivo* experiments. Thus, the present experimental-computational formalism must be considered a first step in developing a systematic approach to calibrating model parameters from experimental data.

In spite of the above limitations, the model can be adapted to account for all the previously mentioned complexities of the tumor microenvironment in parallel with more physiological tumor platforms, to predict cancer growth and response to various therapeutics. Indeed, we note that we have developed substantially more experimental systems^47–49,52^, that take into account the limitations described earlier, and comprehensive models^3–11,53^ suitable for both *in vitro* and *in vivo* application.

## Electronic supplementary material

Supplementary Material
