# Supplementary material for: Calibration of Multi-Parameter Models of Avascular Tumor Growth Using Time Resolved Microscopy Data
Source: Sci Rep. 2018 Sep 28;8:14558. doi: 10.1038/s41598-018-32347-9 (PMC6162291; doi:10.1038/s41598-018-32347-9)
Supplement: Supplementary file 1 — Supplementary Material [file 41598_2018_32347_MOESM1_ESM.pdf]

Calibration of Multi-Parameter Models of Avascular Tumor Growth Using Time Resolved Microscopy Data

E. A. B. F. Lima[1,*], N. Ghousifam[2,+], A. Ozkan[2,+], J. T. Oden[1], A. Shahmoradi[3,4], M. N. Rylander[2,3], B. Wohlmuth[5], and T. E. Yankeelov[1,3,6,7,8]

[1]Institute for Computational Engineering and Sciences, The University of Texas at Austin, Austin, 78712, USA
[2]Department of Mechanical Engineering, The University of Texas at Austin, Austin, 78712, USA
[3]Department of Biomedical Engineering, The University of Texas at Austin, Austin, 78712, USA
[4]Department of Neurology, Dell Medical School, The University of Texas at Austin, Austin, 78712, USA
[5]Department of Mathematics, Technical University of Munich, Garching, 85748, Germany
[6]Department of Diagnostic Medicine, The University of Texas at Austin, Austin, 78712, USA
[7]Department of Oncology, The University of Texas at Austin, Austin, 78712, USA
[8]Livestrong Cancer Institutes, Dell Medical School, The University of Texas at Austin, Austin, 78712, USA
[*]lima@ices.utexas.edu
[+]these authors contributed equally to this work

Supplementary Material

Let $\mathscr{M}_i$ be a mathematical model, among a set $\mathscr{M}$ of parametric model classes, characterized as

$$\mathscr{M}_m : A_m(\theta_m, \phi_m, S_m) = 0, \qquad 1 \leq m \leq 3, \tag{1}$$

where A_m denotes the operator defining the model of interest, $\theta_m \in \Theta_m$ is the vector of parameters for model $\mathscr{M}_m$ with Θ_m being the corresponding parameter space of the model, ϕ_m is the vector of solution of the forward problem, and S_m is the scenario in which the model is applied. In this work, ϕ_m, θ_m, and A_m, $1 \leq m \leq 3$, are defined as

$$\phi_1 = \{\phi_V\}, \qquad \theta_1 = \{\lambda_{apop}\}, \qquad A_1(\theta_1, \phi_1) = \frac{d\phi_V}{dt} = -\lambda_{apop}\phi_V, \tag{2}$$

$$\phi_2 = \{\phi_V\}, \qquad \theta_2 = \{\lambda_{apop}, \lambda_{prol}, K\}, \qquad A_2(\theta_2, \phi_2) = \frac{d\phi_V}{dt} = \lambda_{prol}\phi_\sigma\phi_V\left(1 - \frac{\phi_V}{K}\right) - \lambda_{apop}\phi_V, \tag{3}$$

$$\phi_3 = \{\phi_T, \phi_N\}, \quad \theta_3 = \{\lambda_{apop}, \lambda_{prol}, K, \lambda_{VN}\}, \quad A_3(\theta_3, \phi_3) = \begin{cases} \dfrac{d\phi_T}{dt} &= \lambda_{prol}\phi_\sigma(\phi_T - \phi_N)\left(1 - \dfrac{\phi_T}{K}\right) \\ &- \lambda_{apop}(\phi_T - \phi_N), \\ \dfrac{d\phi_N}{dt} &= \lambda_{VN}(\phi_T - \phi_N). \end{cases} \tag{4}$$

The scenarios are developed such that their complexity builds upon the previous scenarios, and they are defined as

- S_1 - calibration of the apoptosis rate: the cells are treated with Mitomycin C to inhibit cell proliferation;
- S_2 - calibration of the proliferation rate: the cells are not treated with Mitomycin C and are allowed to proliferate;
- S_3 - calibration of the necrosis rate: the viability of the cells is measured under different nutrient availability.

The observational data collected in S_m are denoted y_m. Moreover, the predictions of y_m by model $\mathscr{M}_m$ are denoted $d_m(\theta_m)$. Based on the assumptions of a Gaussian noise-inadequacy model and independent and identically distributed samples of experimental data, the likelihood function is constructed as:

$$\pi(y_m|\theta_m) = \prod_{j=1}^{\mathscr{N}_t}\prod_{i=1}^{\mathscr{N}_r} \frac{1}{\sqrt{2\pi\sigma^2}} e^{-\frac{(y_m^{ij} - d_m^j(\theta_m))^2}{2\sigma^2}}, \tag{5}$$

where $\mathscr{N}_t$ is the number of days measured, $\mathscr{N}_r$ is the number of replicates per day, and σ is an hyperparameter (the standard deviation for the Gaussian) related to the size of the noise.

The Bayesian update in model parameters θ_m is furnished by the posterior probability density $\pi(\theta_m|y_m)$

$$\pi(\theta_m|y_m) = \frac{\pi(y_m|\theta_m)\pi(\theta_m)}{\pi(y_m)}, \tag{6}$$

where the denominator, called the model evidence, is the normalizing factor

$$\pi(y_m) = \int_{\Theta_m} \pi(y_m|\theta_m)\pi(\theta_m)d\theta_m. \tag{7}$$

In Eq. (6), $\pi(\theta_m)$ denote the prior probability densities on parameters θ_m. The posterior is computed through a multilevel Monte Carlo method that is available in the library QUESO[?]. During the simulation, each level of this Monte Carlo algorithm is composed by 200,000 samples. The calibration of the models involve the following steps:

1. Initially, to calibrate $\mathscr{M}_1$ and compute the posterior $\pi(\theta_1|y_1)$, as no prior information is known about the parameter λ_{apop}, it is assumed an uniform prior $\lambda_{apop} \sim \mathscr{U}(a^p, b^p)$, where a^p and b^p are the bounds of the parameter.
2. To calibrate $\mathscr{M}_2$, the apoptosis prior is $\lambda_{apop} \sim \pi(\theta_1|y_1)$. The priors for the parameters λ_{prol} and K are assumed as uniform. Using Eq. (6), the posterior $\pi(\theta_2|y_2)$ is computed.
3. Finally, to calibrate $\mathscr{M}_3$, $\pi(\theta_2|y_2)$ is used as prior to λ_{apop}, λ_{prol}, and K. The λ_{VN} prior is assumed as uniform when computing the posterior $\pi(\theta_3|y_3)$.

After each step, we sample 200,000 values from the posterior and run the forward model. The difference between the forward model solution and the observational data is given by

$$\mathbb{d}_t(F_t, S_t) = \frac{\int_0^\infty |F_t(\phi_V) - S_t(\phi_V)|\,\mathrm{d}\phi_V}{\bar{y}_t}, \tag{8}$$

where $F_t(\phi_V)$ and $S_t(\phi_V)$ are the cumulative distribution functions (cdfs) for the model and the observational data at day t, and $\bar{y}_t$ is the mean tumor volume fraction of the four biological replicates. An illustration of this metric is presented in Figure 1, where the model $\mathscr{M}_1$ was calibrated. The experimental data cdf is represented by a step function in blue, while the red line represents the cdf of the solution of the calibrated model $\mathscr{M}_1$. The green area represents the numerator of Eq. (8), which, in this case, represents a difference of 0.012 in viable tumor volume fraction. The denominator at day 3 in S_1 is 0.063, leading to $\mathbb{d}_t(F_t, S_t) = 0.19$. This result means that the distance between the two cdfs is 19% of the average volume fraction measured at day 3.

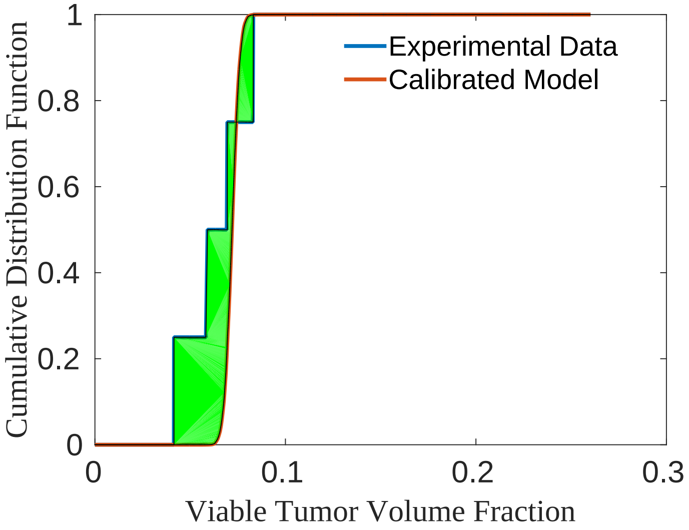

Figure 1. In blue, the cumulative distribution function (cdf) of the experimental data measured at day 3. The cdf is built as a step function with the four replicates measured. In red, the cdf of the forward model computed using 200,000 samples of the calibrated parameters. In green the distance between the calibrated model and the experimental data. The average distance between the data and the model viable tumor volume fraction is 0.012, which corresponds to 19% of the average value measured experimentally.
